# Content of PAHs in soil of a hazel orchard depending on the method of weed control

**DOI:** 10.1007/s10661-018-6812-2

**Published:** 2018-06-25

**Authors:** S. J. Krzebietke, J. Wierzbowska, P. J. Żarczyński, S. Sienkiewicz, M. Bosiacki, B. Markuszewski, A. Nogalska, E. Mackiewicz-Walec

**Affiliations:** 1 Department of Agricultural Chemistry and Environment Protection, University of Warmia and Mazury in Olsztyn, Olsztyn, Poland; 2 Department of Horticulture, University of Warmia and Mazury in Olsztyn, Olsztyn, Poland; 3 Department of Horticultural Plant Nutrition, Poznań University of Life Science, Poznań, Poland

**Keywords:** PAHs, Soil mulching, Mulch fabric, Bark chips, Sawdust, Manure compost

## Abstract

PAHs belong to persistent organic pollutants (POPs) found in the natural environment. They eventually accumulate in the highest quantities in soil. The purpose of this study has been to determine quantities of PAHs in soil depending on the method applied to control weeds in rows of a 4-year plantation of hazel (mulch fabric, bark chips, sawdust, manure compost, bare fallow, chemical fallow, grass sward). The highest concentration of PAHs (16 PAHs) was found in soil kept as bare fallow. The second most abundant concentration of these compounds was determined in soil under grass sward, followed by soil under sawdust, chemical fallow, and fabric. Less of these compounds accumulated in soil mulched with bark chips. The best method for protection of orchard soil against the accumulation of unwanted and toxic PAHs was mulching with manure compost. In most cases, lower concentrations of PAHs (total 16) were found in the subsoil (30–60 cm) than in the topmost soil layer, except the soil covered with mulch fabric, where fourfold more PAHs accumulated.

## Introduction

Polycyclic aromatic hydrocarbons belong to toxic and carcinogenic compounds. They appear in practically all elements of the natural environment exposed to human impact. They can arrive into the environment components (soils, atmosphere, and water) from natural and anthropogenic sources (Jancewicz et al. 2015; Tsibart and Gennadiev 2013). They are generated by incomplete coal combustion, contamination of soil with petrol or diesel oil (Kucharski et al. 2010; Borowik and Wyszkowska 2018b), but changes in their concentration have also been noted due to natural mineralization of organic matter in soil (Mrozik et al. 2003; Wyszkowski and Ziółkowska 2013).

Most research on transformations and accumulation of PAHs in soil deals with the management of organic waste which contain toxic compounds, especially sewage (Wild et al. 1991; Oleszczuk and Baran 2005; Oleszczuk 2008; Oleszczuk 2009a; Oleszczuk 2009b), sludge (Vácha et al. 2010), and wastewater (Khan et al. 2008). Polycyclic aromatic hydrocarbons are hardly mobile, practically insoluble in water, characterized by high affinity to organic matter and do not undergo physical or chemical degradation, although they can be transformed through photochemical reactions, giving rise to diols, phenols, and aldehydes as well as epoxides (Suszek 2007). According to Sinkkonen and Paasivirta (2000), transformations of PAHs in soil can occur only down to a soil depth of 1 mm. Higher soil temperature favors photodegradation of so-called light PAHs and formation of toxic metabolites of “heavy” PAHs in soil (Marquès et al. 2016). Only microorganisms and enzymes are capable of breaking down chains of benzene rings (Miles and Doucette 2001; Mrozik et al. 2003; Baran and Oleszczuk 2002; Oleszczuk and Baran 2006; Klimkowicz-Pawlas and Maliszewska-Kordybach, 2003). The biological degradation of PAHs can also be carried out by fungi (Borowik et al. 2017; Gąsecka et al. 2012). In general, PAHs cause an increase in the abundance of microorganisms and in the activity of soil enzymes (Borowik and Wyszkowska 2018a, Borowik and Wyszkowska 2018b, Borowik et al. 2017), but are phytotoxic to a *Lepidium sativum*, *Sorghum saccharatum and Sinapis alba* (Lipińska et al. 2015), *Avena sativa* (Borowik and Wyszkowska 2018b, Wyszkowska et al. 2015), and *Zea mays* (Borowik and Wyszkowska 2018a). Higher activity of soil enzymes contributes to a more intensive degradation of hydrocarbons (Borowik and Wyszkowska 2018a, Borowik et al. 2018), but it may have a negative effect on microbial diversity (Borowik et al. 2017, Borowik and Wyszkowska 2018a).

The purpose of this study has been to determine the content of PAHs depending on the method applied to control weeds in rows of a 4-year hazel plantation (mulch fabric, bark chips, sawdust, manure compost, bare fallow, chemical fallow, and grass sward).

## Material and methods

**Fig. 1:**
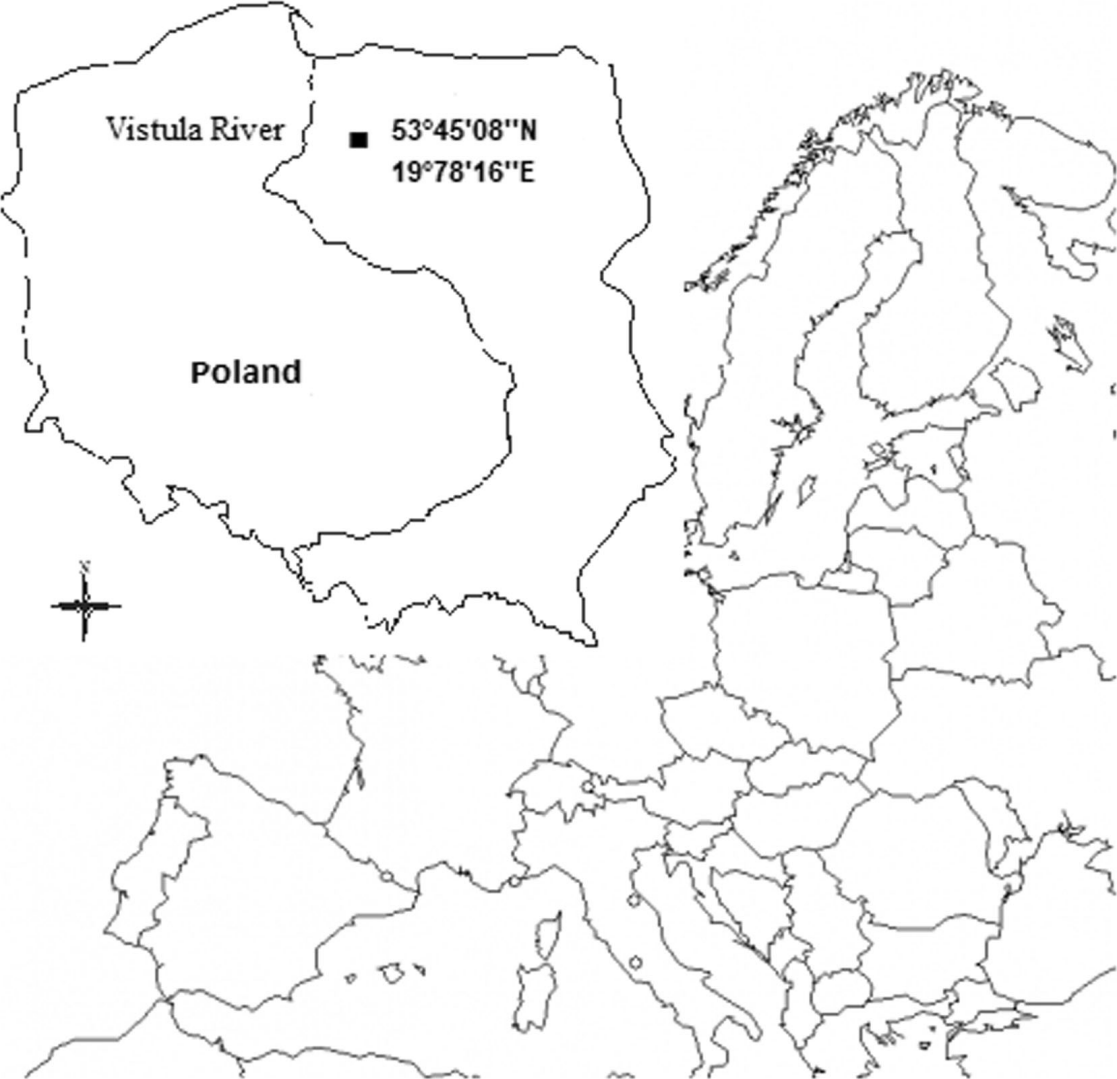
Location of the experiment

The experiment with different mulching techniques for controlling weeds in a hazel orchard was set up in Tuszewo 53° 47′ 02″ N, 19° 78′ 16″ E near Lubawa (the Province of Warmia and Mazury, Poland) in 2006 (Fig. 1). The experiment comprised seven ways of weed control (bark chips, sawdust, composted manure, chemical fallow, bare fallow, grass sward, mulch fabric). Mulching was started in the second year of growing hazel trees. Manure compost tested in the experiment originated from a poultry farm and was applied after 1 year composting with added straw (10%), in a dose of 20 t/ha of row. Two varieties of hazelnut were grown (Kataloński and Halle). All the plant protection and cultivation treatments were performed according to the recommendations contained in the *Fruit Plant Protection Program* 2012 and a set of guidelines (*Zdyb* 2012)*.*

Soil samples for analyses were collected from the 0–30 cm and 30–60 cm layers twice, in the autumn of 2012 and 2013, after the growing season had terminated (Table 1). Extractions of N-NH_4_, N-NO_3_, P, K, Ca, Mg, S-SO_4_, Na, and Cl were carried out in 0.03 M CH_3_COOH. After extraction, the following determinations were made: N-NH_4_ and N-NO_3_ by potentiometric methods (Microprocessor pH/ION Meter pMX 3000 WTW, Germany); S-SO_4_—nephelometric with BaCl_2_; Cl—nephelometric with AgNO_3_; P—colorimetric method with ammonium vanado-molybdate (UV—1201 V spectrophotometer, Shimadzu Corporation Kyoto, Japan) K, Ca, Na—by atomic emission spectrometry (AES) (BWB Technologies UK Ltd.); and Mg—by atomic spectrometry absorption (ASA) (AAS1, Carl Zeiss Jena, Germany). pHH2O was determined in water solution by the potentiometric method, and EC by the conductometric method (Nowosielski 1974; Komosa, Stafecka 2002).

**Table 1:** Mean levels of nutrients in soil of a hazel orchard in 2012–2013, depending on the experimental treatments

No.	Treatments	Layers	S-SO_4_	Cl	P	N-NH_4_	N-NO_3_	Ca	Mg	K	Na	pH	EC
			mg/dm^3^ of soil									H_2_O	mS/cm
1	Bare fallow	0–30 cm	25.1	13.6	69.8	7.0	0.8	958.2	57.1	190.4	19.6	6.70	0.07
2	Grass sward		27.7	11.0	29.6	4.6	1.3	747.4	50.5	138.9	12.7	4.90	0.01
3	Sawdust		25.8	16.0	31.9	7.2	1.9	595.2	21.3	137.5	10.2	6.30	0.03
4	Manure compost		43.6	13.6	104.5	10.8	27.6	918.9	199.5	352.6	11.2	6.00	0.20
5	Bark chips		38.6	31.3	72.0	10.2	2.1	698.0	27.4	145.0	17.6	6.33	0.03
6	Chemical fallow		23.2	8.6	65.5	11.7	2.4	949.9	36.9	116.8	14.2	5.80	0.05
7	Fabric		24.6	85.0	38.7	6.0	28.1	722.7	37.2	156.0	14.6	5.60	0.02
1	Bare fallow	30–60 cm	29.2	21.1	43.1	10.4	1.9	940.6	47.15	77.5	9.9	6.20	0.05
2	Grass sward		17.1	5.6	21.9	3.8	1.1	574.6	17.6	25.9	1.9	5.22	0.02
3	Sawdust		19.8	6.4	20.1	6.4	1.0	821.4	18.9	38.2	6.2	6.00	0.04
4	Manure compost		18.6	10.6	97.5	10.2	2.4	624.0	74.6	320.6	2.7	5.50	0.10
5	Bark chips		16.3	5.6	15.3	9.6	1.0	747.1	20.1	33.3	2.7	6.10	0.02
6	Chemical fallow		29.6	7.6	75.2	18.8	1.9	917.7	35.7	55.4	6.5	5.60	0.05
7	Fabric		25.8	8.0	24.8	6.2	1.8	722.7	12.6	28.4	7.0	5.30	0.02

Analysis of the content of PAHs in air-dry soil passed through a 2-mm mesh size sieve, additionally ground in an agate grinder to the size of < 0.5 mm (MN Appl. No. 301320 n.d.) was performed after 1 h extraction of 20 g of soil with 30 cm^3^ of acetonitrile in three replications using an ultrasonic washer. SPE columns of the capacity of 6 cm^3^ containing 2 g of Florisil were conditioned with methanol. The extract (10 cm^3^) was decanted and purified in an MPW-350R centrifuge and an SPE station. Ten cubic centimeters of methanol was used to rinse polar compounds off the sorbent bed. Afterwards, the extract was concentrated to 0.2 cm^3^ in the neutral gas (nitrogen) atmosphere. The samples prepared as described above were submitted to determination of selected PAHs with the (GC-MS) technique using FID detectors on a Rxi-5 ms column 30 m long, 0.25 mm ID, and 0.25 μm. The carrier gases were He at constant flow (3 cm^3^/min) as well as H_2_, air, and N_2_ flowing at 35, 350, and 30 cm^3^/min, respectively. The following temperature regime was set: 0–100 °C—0.2 min, 50 °C/min–143 °C—1.5 min, 8 °C/min–180 °C—0.4 min, 100 °C/min–210 °C—1.5 min, 10 °C/min–300 °C—5 min = 23.39 min. The temperature at the detectors was set at 340 °C, and the temperature of a splitless injector was 250 °C. Determinations were made with reference to a model solution by Restek Corporation containing a mix of 16 PAHs (naphthalene, acenaphthylene, acenaphthene, fluorene, phenanthrene, anthracene, fluoranthene, pyrene, benzo(a)anthracene, chrysene, benzo(b)fluoranthene, benzo(k)fluoranthene, benzo(a)pyrene, indeno(1,2,3-cd)pyrene, dibenzo(a,h)anthracene, benzo(g,h,i)perylene) in a concentration of 2000 μg/cm^3^ of each of the components. Working calibration standards were set at 5, 10, 20, 50, 100, and 200 μg/cm^3^ of each of the components. The recovery of compounds in soil ranged from 84 to 93%, and the respective result was included in the computations for each of the compounds separately. Statistical calculations comprised one-factorial analysis of variance using Statistica v.10 software as well as cluster analysis and main component analysis with MVPS v. 3.1 software.

## Results and discussion

**Fig. 2:**
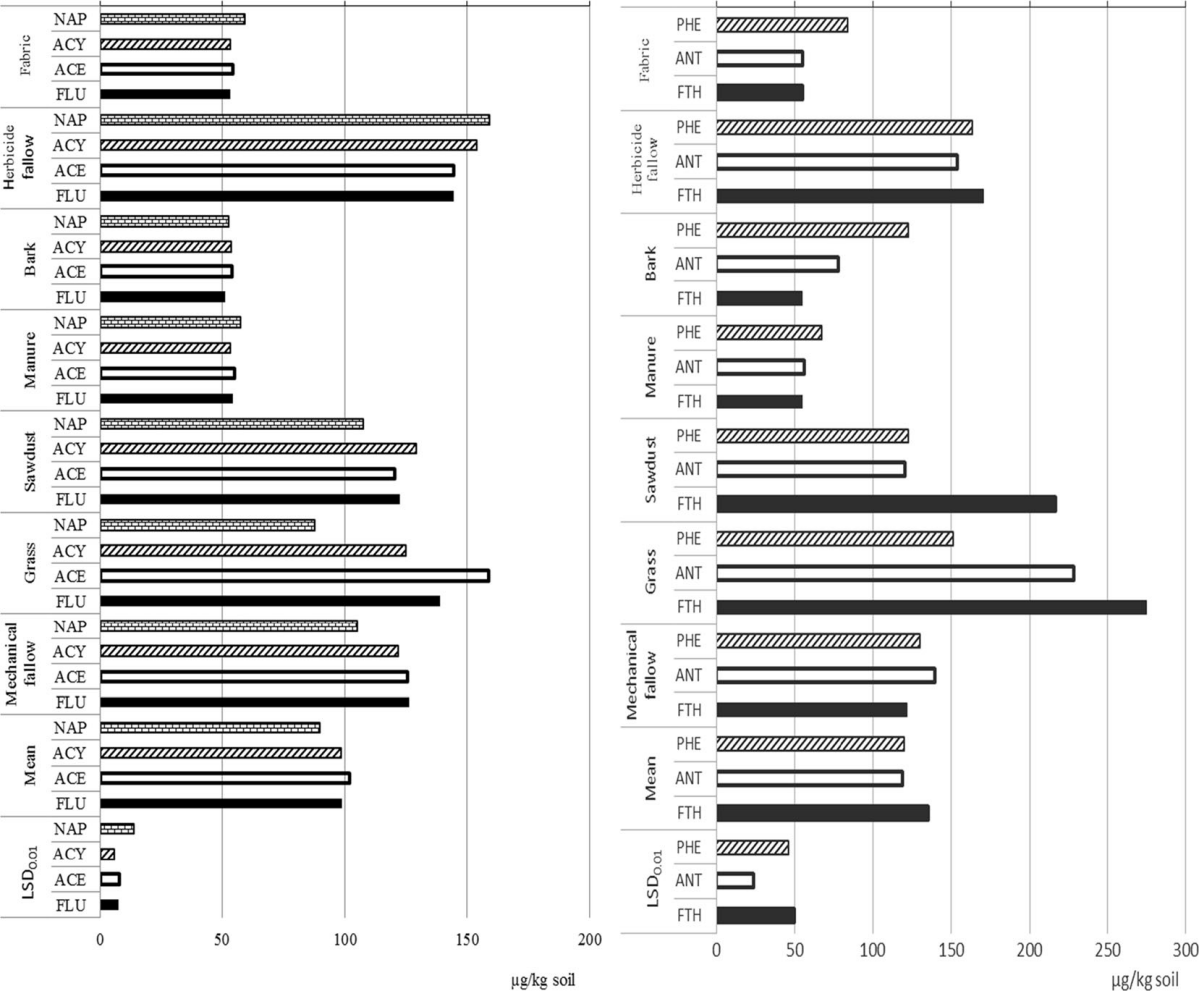
Average content of selected PAHs (significant differences) from 2012/2013 depending on the method of maintaining rows in a hazel orchard in the soil layer 0–30 cm. NAP naphthalene, ACY acenaphthylene, ACE acenaphthene, FLU fluorene, PHE phenanthrene, ANT anthracene, FTH fluoranthene, LSD_0.05_ least significant differences

The concentration of two- and three-ring aromatic hydrocarbons dominated the accumulation of 16 PAHs in soil (Fig. 2). The soil from the treatments where mulch fabric, bark chips, and manure compost had been applied contained the smallest amounts of aromatic hydrocarbons composed of two and three benzene rings (naphthalene, acenaphthylene, acenaphthene, fluorene). The content of these compounds was quite uniform and ranged between 50 to 60 μg/kg of soil (Fig. 2). When rows between trees had been kept as mechanical fallow or mulched with sawdust, the accumulation of the above compounds was twice as high, but with a lower amount of naphthalene. Grass sward strongly differentiated the amounts of two-ring PAHs, from 87.5 (naphthalene) to 158.7 μg/kg of soil (acenaphthene). The highest concentrations of NAP, ACY, and FLU were determined in soil under herbicide fallow (Fig. 2).

Manure compost, bark chips, and fabric used for mulching rows resulted in similar amounts of soil-accumulated fluoranthene, one of the members of PAHs possessing three benzene rings. The highest accumulation of fluoranthene (274.6 μg/kg of soil) was detected in soil under grass sward and mulched with sawdust (217.15 μg/kg). In the soil layer 0–30 cm, the two-ring hydrocarbon naphthalene under herbicide fallow exceeded the threshold concentration set in the Regulation of the Minister for the Environment of 5 September 2016 on how to assess the pollution of the earth surface (Dz. U. of 2016 item 1395) by an average of 57.2%, under sawdust by 8%, and under mechanical fallow by 5.3%, whereas anthracene was by an average 37.5% higher than the limit value.

**Fig. 3:**
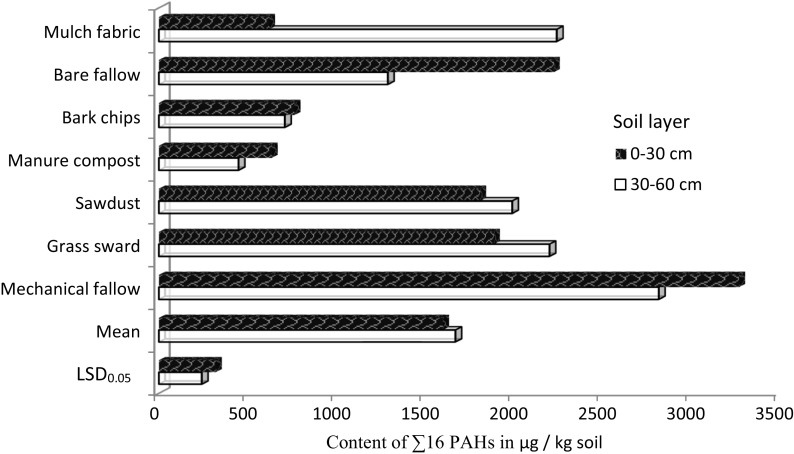
Average content of ∑16 PAHs in the 0–30 cm and 30–60 cm soil layer in a row of a hazel orchard depending on the method of weed control in 2012–2013. LSD_0.05_ least significant differences

The content of PAHs (the total of 16 compounds) in the 0–30 cm soil layer ranged between 621.1 and 3269.7 μg/kg of soil (Fig. 3). When rows between trees had been covered with fabric or mulched with bark chips or sawdust, the amount of these PAHs was slightly higher than the threshold value of < 600 μg/kg of soil (Kabata-Pendias et al. 1995). The binding law in this regard is the Regulation of the Minister of the Environment of 5 September 2016 on the conduct of the assessment of surface contamination of land (Dz. U. of 2016 item 1395). The regulation imposes an obligation to determine 10 compounds representing PAHs: naphthalene, anthracene, chrysene, benzo(a)anthracene, dibenzo(a,h)pyrene, benzo(b)fluorene, benzo(k)fluorene, benzo(ghi)perylene, indeno(1,2,3-c,d)pyrene, as well as raising the threshold total amount of the above compounds to 1400 μg/kg in arable soil. In the experiment reported in this paper, the sum of 10 PAHs was not found to have exceeded the limit value. Herbicide fallow had led to an over threefold higher accumulation of these harmful compounds belonging to polycyclic hydrocarbons in soil. The content of PAHs in soil from the treatments with grass sward and sawdust was above the average. With respect to the accumulation of these toxic compounds (soil contamination with PAHs), bare fallow proved to be the worst technology. The pattern of concentrations of 16 PAHs in the subsoil (30–60 cm) under a hazel orchard was completely opposite (Fig. 3). When fabric had been used to control weeds in rows, the content of the analyzed PAHs in the subsoil was 3.6-fold higher than in the arable soil layer; analogous differences reached 1.16-fold for grass sward and 1.1-fold for sawdust. Herbicide and bare fallows as well as manure compost and bark chips resulted in a lower content of these PAHs in the subsoil compared to the 0–30 cm soil layer. The respective differences were 934.1, 451.8, 186.8, and 53.8 μg/kg of soil depending on the mulching method.

**Fig. 4:**
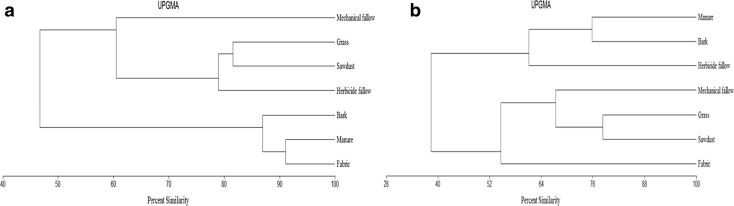
Dendrogram of similarities of the weed control methods in soil in a hazel orchard on accumulation of PAHs in the 0–30 cm (**a**) and in the 30–60 cm (**b**) soil layer

The cluster analysis based on the percent similarities in the concentrations of PAHs in the 0–30 cm soil layer demonstrated high differentiation depending on the materials used for weed control (Fig. 4a). The first group ranking the highest similarity in the PAH content (91%) comprised soil mulched with manure compost and covered with mulch fabric. Soil mulched with bark chips (87%) was the closest to the first group in terms of amounts of PAHs. The second group of similar concentrations of PAHs in soil consisted of the treatments with grass and sawdust (87%) as well as herbicide fallow (78%). The least similar was the content of the total of 16 PAHs in soil under mechanical fallow (60%) versus the soil under grass sward, sawdust, and chemical fallow. In the 30–60 cm soil layer, the accumulation of PAHs and generated clusters was completely different from the ones in the upper soil layer (Fig. 4b). The dendrogram showed the highest degree of similarity in the concentration of the determined PAHs in soil under grass sward and sawdust; less similar was the content of PAHs in the subsoil under bare fallow (65%). The second group comprised treatments with the content of the analyzed xenobiotics determined in the subsoil under manure compost and bark chips used to mulch rows and under herbicide fallow (60%).

The accumulation of these undesirable compounds most often occurs as a result of an inappropriate C:N ratio and inadequate humus content in soil. According to Kabata-Pendis et al. (1995) and Siebielec et al. (2012), the content of humus in soil up to 2% reflects its natural processes of PAH transformations, while at a content of humus above 2%, it is necessary to include correction factors for making comparisons of the content of 13 PAHs in soil. More intensive mineralization processes in soil additionally contribute to a higher accumulation of these compounds in soil. Mechanical eradication treatments on mechanical fallow and low supply of organic matter can stimulate mineralization of organic matter and consequently lead to a higher accumulation of the toxic compounds which belong to polycyclic aromatic hydrocarbons. The cycle of decomposition of certain herbicides is similar to that of some PAHs. When microorganisms have access to carbon from plant protection chemicals, they use it more readily than less easily available carbon from PAHs. This phenomenon most probably contributed to a higher accumulation of these compounds in soil under herbicide fallow. Development of soil microflora able to decompose unwanted compounds primarily depends on the availability of other nutrients, especially nitrogen but also P, K, S, Ca, Mg, and the toxicity of some herbicides towards soil microorganisms active in PAH mineralization (Wyszkowski and Wyszkowska 2005; Maliszewska-Kordybach et al. 2007). Good conditions for the decomposition of PAHs were created by manure compost, with which soil was enriched with most of the basic macro- and microorganisms, essential for the proper growth and development of plants as well as for maintaining microbiological life. Other important aspects are the proper C:N and P ratios, which condition the multiplication of microorganisms, temperature about 30–38 °C, oxygen supply, optimal soil moisture and soil reaction from slightly acid to alkaline (pH 6.5–7.5), and access to light (Mrozik et al. 2003; Yan et al. 2004; Chang Chien et al. 2011). It can be concluded that mulch fabric spread on rows between trees in the orchard raised the soil temperature, which in turn accelerated the growth of microorganisms and consequently depressed the concentration of PAHs in soil. This effect of fabric application appeared only in the surface soil layer. Fourfold more PAHs were found in the subsoil. Exceeding the set threshold levels, especially the third (contaminated soils) and higher ones, of PAHs in the surface soil layer may have impact on the transfer of the toxins to plants, as claimed by Kabata-Pendias et al. (1995). The uptake of PAHs from contaminated soils by plants might subsequently affect their chemical composition, yield, and yield quality (Krzebietke and Sienkiewicz 2010a; Krzebietke and Sienkiewicz 2010b; Gondek et al. 2014).

## Conclusion

1. The highest concentration of polycyclic aromatic hydrocarbons (16 PAHs) was found in soil under bare fallow. With respect to the increasing accumulation of PAHs in the 0–30 cm soil layer, the methods applied to control weeds can be arranged in the following sequence: fabric < manure compost < bark chips < sawdust < grass sward < herbicide fallow < mechanical fallow; and in the 30–60 cm layer: manure compost < bark chips < herbicide fallow < sawdust < grass sward < fabric < mechanical fallow.
2. In most cases, lower concentrations of PAHs were determined in the subsoil (30–60 cm) than in the upper soil layer (0–30 cm), except the treatments with mulch fabric, where the content of PAHs was fourfold higher.
3. Polycyclic aromatic hydrocarbons containing two to four benzene rings in a molecule (naphthalene, acenaphthylene, acenaphthene, fluorene, phenanthrene, anthracene, fluoranthene, pyrene, benzo(a)anthracene and chrysene) were dominant PAHs in the tested soil.
4. The content of the other hydrocarbons, known as heavy ones (benzo(a)pyrene, benzo(b)fluoranthene, benzo(k)fluoranthene, indeno(1,2,3-cd)pyrene, dibenz(a,h)anthracene, benzo(g,h,i)perylene), did not exceed 10 μg/kg of soil.
